# Divergent responses of forage quality indicators to environmental drivers in Eastern Mongolia

**DOI:** 10.3389/fpls.2026.1860377

**Published:** 2026-08-24

**Authors:** Yun Jäschke, Oyunbileg Munkhzul, Uuganjargal Khaliun, Batlai Oyuntsetseg, Thanh-Noi Phan, Shuxin Ji, Lukas Lehnert, Khurelpurev Oyundelger, Christiane M. Ritz, Karsten Wesche

**Affiliations:** 1 Senckenberg Museum for Natural History Görlitz, Senckenberg – Leibniz Institution for Biodiversity and Earth System Research, Görlitz, Germany; 2 Mongolian Society of Mammalogy, Ulaanbaatar, Mongolia; 3 Institute of Geography and Geoecology, Mongolian Academy of Sciences, Ulaanbaatar, Mongolia; 4 Department of Biology, School of Arts and Sciences, National University of Mongolia, Ulaanbaatar, Mongolia; 5 Department of Geography, Ludwig-Maximilians-Universitat Munchen, Munich, Germany; 6 Technische Universitat Dresden, Internationales Hochschulinstitut Zittau, Zittau, Germany; 7 Deutsches Zentrum fur Integrative Biodiversitatsforschung (iDiv) Halle-Jena-Leipzig, Leipzig, Germany

**Keywords:** aboveground biomass, carrying capacity, crude protein yield, digestibility, disturbance, functional composition, plant nitrogen content, steppe rangeland

## Abstract

Forage quality is crucial for determining livestock productivity as well as ecosystem health, and consequently is a critical parameter for sustainable rangeland management. However, most studies concentrate on forage amount alone, and it remains largely unknown to what extent climate, soil, and disturbance impact seasonal forage quality. Across spring, summer and autumn, we conducted a large-scale field study at 63 sites in the eastern Mongolian steppe, arguably the largest intact steppe region under traditional mobile land use. We performed exploratory multivariate analyses to check the correlation structure among forage indicators and environmental factors separately, and then used linear mixed models to assess the impact of season, and major environmental factors, i.e., precipitation, temperature, soil carbon-nitrogen (C-N) ratio, local disturbance, as well as the interaction between precipitation and disturbance on current standing biomass, and selected indicators of forage quality, i.e., community-weighted nitrogen content, crude protein (CP) yield, digestibility and water content of fresh biomass. Results showed a clear seasonal difference in all indicators except digestibility. Plant N contents at the community level declined significantly from 2.4% in spring to 2.1% in summer, then to 1.5% in autumn, while mean CP yield was 104 kg·ha^-^¹in summer and more than halved towards spring. Water content in fresh biomass was highest in summer (54%) and lowest in autumn (30%). Linear modeling revealed that higher precipitation was associated with greater CP yield, while higher disturbance lead to lower CP yield. Temperature was a significant positive predictor of total water content. Digestibility was not sensitive to any predictor tested. We highlight the importance of integrating forage quality and its seasonal dynamics into estimates of grassland carrying capacity.

## Introduction

Rangelands support livelihoods for millions of people worldwide by providing forage for their livestock. Forage availability in terms of forage quantity and quality directly determines the carrying capacity of grasslands and the productivity of livestock, and is thus is of outmost concern for animal husbandry, local livelihoods and ecosystem health. Aboveground biomass (AGB) is often used as an indicator for evaluating impacts of changing climate and land use on integrity and functioning of rangeland ecosystems (e.g., Ojima et al., 2020), either in rangelands globally (Piipponen et al., 2022), or regionally (Yan et al., 2023; Wang et al., 2024a).

Variation in AGB is controlled by grazing, climate (Wu et al., 2021), and soil properties (Reinhart and Vermeire, 2017). Reviews revealed that heavy grazing negatively affected AGB, although the magnitude of grazing effects differed among grassland types in Mongolia (Munkhzul et al., 2021) and China (Wang and Wesche, 2016). Grazing effects on AGB interact with climate (Cao et al., 2024; Ahlborn et al., 2020). Studies described either stable too increasing (Khishigbayar et al., 2015) or highly variable AGB (Wesche et al., 2010) with changing climate, while others reported decreasing AGB in Mongolia and Inner Mongolia (Godde et al., 2020; Wang et al., 2021). Some demonstrated the cumulative effect of overgrazing as a primary contributor to grassland degradation (Hilker et al., 2014; Yang et al., 2016; Zhang et al., 2020), while a debated recent study pointed out that climate rather than livestock number drives most of the productivity (Purevjav et al., 2025).

**Figure 1:**
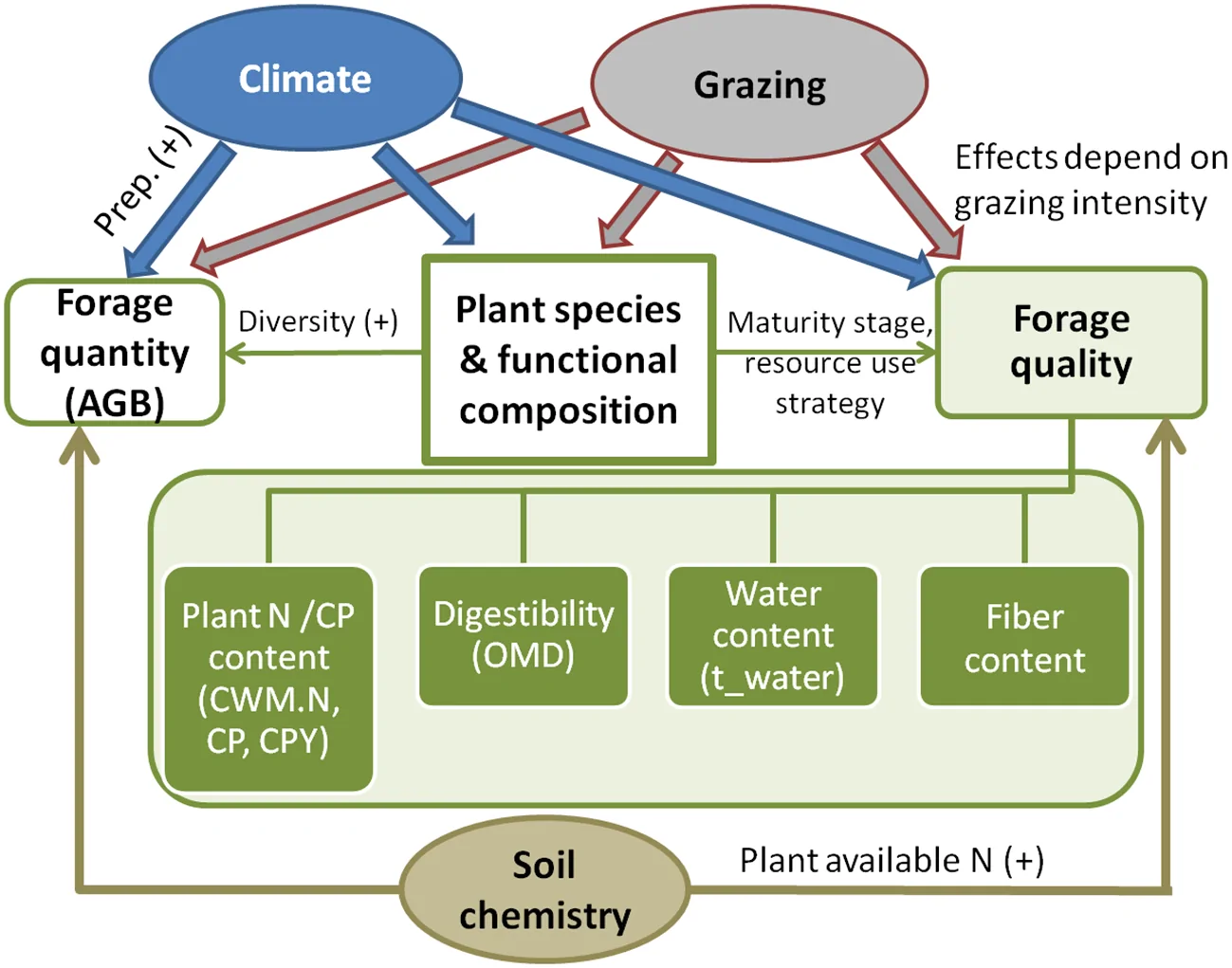
Environmental drivers of forage availability, i.e., forage quantity and quality. Forage quantity is represented by current standing biomass (AGB), and forage quality includes the following aspects: plant nitrogen (N), crude protein (CP) content, digestibility, water content, and fiber content. In the current study, we investigate community-weighted mean N content (CWM.N), mean crude protein content (CP) and yield (CPY), organic matter digestibility based on dung of sheep/goats (OMD), and total water content in fresh biomass (t_water). Fiber content was not investigated. “Prep.” means precipitation. “+” indicates generally positive impact. The potential impact of climate and grazing on forage quality is further summarized in **Table 1**.

Compared to the large number of studies on AGB, forage quality has been rarely evaluated at large spatial scales, or along climatic gradients. Forage values are traditionally measured by wet chemistry methods, i.e., contents of crude fat, nitrogen (N), crude protein (CP), digestible organic matter, and structural carbohydrates, i.e., fiber and lignin (Angerer, 2012). Their importance is shown by a study from Inner Mongolia, where the carrying capacity based on forage biomass was estimated to be 1.5 sheep units per ha, but only 1.3 sheep units per ha based on CP consumption (Zhao et al., 2023). Forage quality is strongly associated with plant functional group composition, as well as with their stage of maturity and resource use strategy, but is also influenced by climate and soil chemistry (**Figure 1**, **Table 1**, Dellar et al., 2018; Perotti et al., 2021). At the global scale, leaf N content is positively correlated with mean annual temperature (Ordoñez et al., 2009). Precipitation was found to positively influence CP content in Inner Mongolian grasslands (Schönbach et al., 2012; Zhao et al., 2023). Organic matter digestibility (OMD) also is crucial for livestock. Higher contents of N (therefore higher CP) indicate better forage quality, while higher fiber contents typically indicate lower digestibility and thus lower forage quality. A meta-analysis of climate manipulation experiments suggested that water stress could increase forage N content and diges-tibility but reduces plant cell-wall (NDF) content (Dumont et al., 2015). Some studies also imply that the response of leaf N content to drought depends on plant functional types (Yan et al., 2019). Forage water content, although rarely investigated, may affect diet selection and intake of dry matter (Pasha et al., 1994; Cain et al., 2017). Forage with high water content can supply livestock and wildlife’s water needs, reducing grazer dependence on drinking water in arid regions (Squires, 1981).

**Table 1:** Summary of the potential impact of climate and grazing on three aspects of forage quality, i.e., plant N/CP content and digestibility; for completeness fiber content is added here but not measured in the present study. ↑, ↓ and ns indicate increase, decrease and non-significant trend respectively.

Environmental Factor	Aspects of forage quality			References
	Plant N/CP content	Digestibility	Fiber content	
High temperature	↑, ↓ or ns	↓ (↑ maturation & cell lignification)	↑	Ordoñez et al., 2009; Bai et al., 2013; Sun et al., 2020; Yoshihara et al. 2022
High precipitation	↑	↑ initially (lush growth)	↓ initially, or ns	Schönbach et al., 2012; Zhao et al., 2023
Drought stress	↑ (concentration effect)	↓ (↑ structural compounds)	↑	Dumont et al., 2015; Yan et al., 2019
Elevated CO_2_	↓	↓ or ns	ns	Milchunas et al., 2005; Dumont et al., 2015
High N fertility	↑	↑ if stays vegetative; ↓ if matures faster	↓ if vegetative; or ↑	Milchunas et al., 2005
Intensified grazing	↑	↑ or ns	↓ or ns	Müller, 2009; Schönbach et al., 2009; Miao et al., 2015; Jamsranjav et al., 2018

Mongolian grasslands represent the last intact steppe ecosystem with significant biodiversity under traditional nomadic land use (Batsaikhan et al., 2014). Natural forage is the only major source of feed for livestock in Mongolia, where the livestock sector accounts for over 80 percent of the gross agriculture outputs (FAO, 2023). However, with rapid changes in social systems and climate warming, grassland degradation has become a critical issue (Fernández-Giménez et al., 2017; Sainnemekh et al., 2022). In spite of the controversial discussion on livestock impact on AGB in Mongolia, little information is available on forage quality, except for some short reports of chemical contents in Mongolian language (Damiran and Sainkhuu, 2016) and a few studies related to wild gazelle (Olson et al., 2010). Simulated warming was shown to increase fiber content and decrease digestibility (Yoshihara et al., 2022). A large-scale study on winter pastures (Jamsranjav et al., 2018) suggested higher CP contents under intensive grazing in typical steppes. This is in line with the results from ecologically similar typical steppes of Inner Mongolia (Fanselow et al., 2011; Hoffmann et al., 2016), showing increased forage CP contents with increased grazing intensity. However, whether forage quality changes along large climate and disturbance gradients on Mongolian rangelands is still unknown.

Seasonal patterns in forage availability are also important for pasture management, because growing season in Mongolia is very short and both spring and autumn differ strongly from the short summer phase. This is of high relevance for grazers, as they strongly depend on the first forage after typically harsh winters. Studies from other regions suggest that forage quality varies among seasons. For example, a study from *Festuca* grasslands in Canada suggests that forb CP and fiber content showed opposite trends during the growing season, which is related to physiological stage and degree of senescence (Vera-Velez and Lamb, 2022). Crude protein content was shown to be highest in the early growing season (June), but lowest at the end of the growing season (September) in typical steppe of Inner Mongolia (Ren et al., 2016a), while NDF increased along seasons in alpine meadow in Qinghai Tibetan Plateau (Yao et al., 2022) as wells as in typical steppe in Inner Mongolia (Müller et al., 2014). Additionally, seasonal rainfall patterns (timing and amount) affected vegetation composition, plant height, forage nutrition, and thus livestock conditions (Yoshihara et al., 2024). It is thus essential to consider grassland condition in other seasons besides summer for estimating carrying capacity (Yan et al., 2023). Moreover, the physiological state of the plant and the physical environment change dramatically throughout the year, so the sensitivity of plants to grazing, climate or soil condition may differ seasonally. However, seasonal change of forage availability has only been studied at single site level (Ren et al., 2016a), and no information is available along larger scales in the Mongolian grasslands.

**Table 1** gives a synoptic overview of a number of key studies in the field, and shows inconsistent responses of different facets of forage quality to climate and grazing. Particularly, we found that grazing impact depends on the exact indicator of forage quality, i.e., plant N/CP content generally increased as grazing intensified, while digestibility may increase or did remain unchanged, and fiber content decreased or did not change. In the current study, we focus on impact of precipitation, temperature, soil fertility and grazing on the following indicators of forage quality, i.e., community-weighted plant N content (CWM.N), CP yield (CPY, i.e., CP content multiplied by current standing biomass) and digestibility of organic matter (OMD). Additionally, we are also interested in the pattern of water content of fresh biomass (t_water), on which no previous study has focused. Our study region covers large gradients of climate and local disturbance caused by livestock grazing in the central and eastern Mongolian steppe, and we sampled during spring, summer, and autumn. Our aim is to reveal spatial and seasonal patterns of forage quality (refer to the above four indicators in the following) and to evaluate the relative effects of climate, soil chemistry, and grazing disturbance. Our major research questions are the following: 1) How does forage quality change among three seasons? 2) Does precipitation have positive impact on plant N/CP content and digestibility, and how does temperature influence them? 3) How do different indicators of forage quality change along a livestock grazing gradient represented by distance to settlement center? 4) Does the impact of grazing and climate on forage quality change among the three seasons?

## Methods

### Study area and time

**Figure 2:**
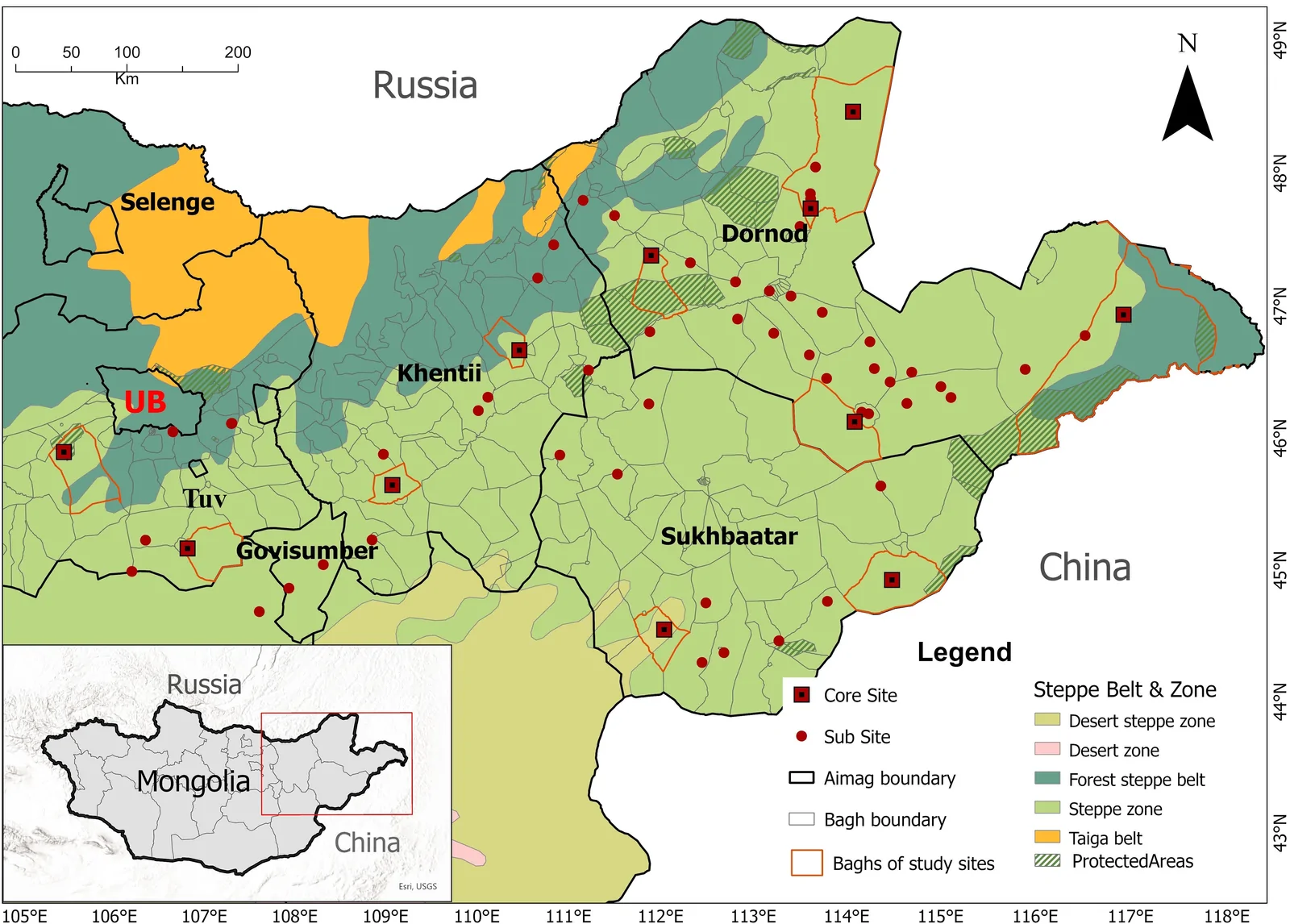
Study region and sampling sites in eastern Mongolia. UB represents Ulaanbaatar, the capital city of Mongolia. Aimag is the highest level of administrative division in Mongolia, equivalent to a province, and bagh is the smallest administrative unit, functioning as a local governance unit.

We conducted our study at 11 core sites and 52 satellite sites (a total of 63 sites) across four *aimags* (the highest level of administrative division in Mongolia, equivalent to a province) from central to eastern Mongolia, i.e., Tuv, Khentii, Dornod, and Sukhbaatar. We covered large environmental gradients (**Figure 2**) in a generally dry continental climate with long, cold winters and short, relatively warm summers, during which most of rainfall occurs (Pfeiffer et al., 2018). The mean annual precipitation (MAP) ranges from 180 to 340 mm, and mean annual temperature (MAT) ranges from -2.2 to 2.3°C (Fick and Hijmans, 2017). Our study region represents the largest steppe type in Mongolia, i.e., the typical/dry steppe zone, situated between the northern and eastern forest steppe belt and the southern desert steppe zone (Pfeiffer et al., 2020). The most dominant plant species include grasses such as *Stipa krylovii*, *S. grandis, Leymus chinensis*, *Cleistogenes squarrosa*, and sedge species like *Carex stenophylla*, subshrubs such as *Artemisia frigida*, *A. adamsii*, and forb species including *Allium polyrhizum*, *Convolvulus ammannii*, *Chenopodium* spp., and *Astragalus* spp.

In mid-July and August (i.e., peak growing season) of 2019 and 2020, we collected summer samples from all core sites and satellite sites. Autumn sampling was conducted from mid- to end of September in both years for those sites we had summer samples for, while spring sampling could only be conducted at end of May in 2020. Due to the travel restrictions during the COVID-19 pandemic, spring sampling was impossible for about 30 sites in the southern part of the study region. Spring and autumn samples were also missing for some sites due to unexpected weather or other travel problems. However, autumn and spring sampling sites still represent all four aimags and cover the same moisture gradient as summer sampling sites, i.e., MAP ranges 180-340 mm, only the MAT gradient is somewhat shorter in the spring samples (-2.2-1.2°C).

It was not feasible to sample all sites within a year given the large spatial distance between sites. However, Mongolian steppes are dominated by perennial plants (Pfeiffer et al., 2018), and moisture condition of both years were similar (Oyundelger et al., 2026), so inter-annual difference in plant community composition should be relatively small. Finally, we had sampled 63 sites in summer, 46 sites re-sampled in autumn, and 28 sites in spring.

### Investigation on plant species and functional composition

We established around 20 vegetation plots (10 m × 10 m) at each core site. For each plot, we assessed plant species and functional composition using a modified Braun–Blanquet approach and visually estimated the percentage of cover for each plant species using modified a Londo scale (Londo, 1976). Sampling at core sites initially aimed at detailed analyses of local disturbance (Jäschke et al., 2026). We amended this with samples from plots single at spatially independent satellite site, in order to more comprehensively cover the large-scale climate and disturbance conditions. To allow comparison, we calculated mean and median values of characteristics of plant community composition and indicators for forage quality (see the following sections) for each core site. Mean values were similar to median values and since our data were not skewed, we thus analyzed means together with single plot-level data from satellite sites.

### Analysis of current standing biomass

Next to each vegetation plot, we collected AGB, i.e., current standing biomass and NOT absolute productivity, from one randomly selected quadrat (1 m × 1 m) at each study site. In each quadrat, living and standing dead aboveground plant material was clipped at ground level. Plant material that had started to wither but was not fully dead, i.e., still partly green, was kept because it still is forage and might be the only source in autumn. AGB was separated into four major functional groups, i.e., shrubs, subshrubs, graminoids (grasses and sedges), and forbs. Subshrubs (chamaephytes) are perennial plants with woody stems except for the terminal parts, which die back annually, such as *Artemisia frigida*, *A. adamsii*, *Kochia prostrata*, and some *Ephedera* spp. Typical shrubs include *Caragana stenophylla*, *C. leucophloea*, and C. *microphylla*, with individuals often lacking large canopies and being below 20 cm in height. From each quadrat, before we cut the AGB, we also recorded vascular plant species richness (sR) and visually estimated the total canopy cover of vegetation (tCov) and the proportion of cover of each functional group. A physical frame of 1 m × 1 m was laid down and we visually grouped plant material into proportional compartments. As AGB samples were collected from non-fenced areas, they only represent residue standing biomass at the time of sampling after potential previous grazing, rather than primary productivity or total forage production. The prior biomass consumption could not be accounted for in the current sampling design. After sorting out stone and litter from AGB, fresh weight was determined in the field first (except for two core sites in 2019) and then taken back to the lab. Samples were then oven-dried at 65 °C for 48 h and weighed again.

### Analyses of forage quality

Air dried biomass samples from different functional groups were cut manually into ca. 1 cm length, and then filled into a ball mill (Retsch MM 400, Retsch GmbH, Germany) for fine grinding to prepare measurements of total C and N contents with a CN analyzer (Vario EL III, Elementar, Germany). For each plot, we calculated the community-weighted means of the plant N content (CWM.N, %), by weighting the N content of each functional group by their respective proportion of the dry biomass weight from the total dry biomass weight. We then calculated the CP content (%) through multiplication by a factor of 6.25 with N content (Hames et al., 2008), assuming that all nitrogen is protein-derived and that protein contains 16% N. As such, we could not account for non-protein N. We also further multiplied CP by AGB to obtain current CP standing crop (CPY, kg·ha^-^¹). Water content was calculated based on the difference of fresh and oven-dried weight of the total AGB.

Organic matter digestibility (OMD) can be estimated *in vitro* using ruminal fluid or enzymatic methods independent of animals but combined with *in vivo* experiments for validation of equations (Schiborra, 2008). Digestibility of forage ingested can also be based on fecal CP content and a calibration established with *in vivo* determined digestibility (Lv et al., 2019; Pringle et al., 2021). We used the later method because it has been applied in Inner Mongolian steppes (Schiborra, 2008; Müller, 2009). In an area surrounding the biomass plot, we collected dung samples of all major livestock groups, i.e., cattle, sheep and goats, and horses. Dung samples were air-dried in the field and taken back to the lab. To determine the organic matter (OM) content, 0.5 g of each dung sample were oven-dried overnight at 105 °C and then ignited at 550 °C for three hours in a muffle furnace (L 24/11/B180, Nabertherm GmbH, Germany) to obtain the weight of ash. OM contents (g·kg^-^¹) were calculated as the difference between the dry weight of the sample and the ash after incineration. The total N content of dung samples was measured with the CN analyzer as described above, and CP content is derived as N content × 6.25. We estimated OMD for the dominant livestock species, i.e., sheep/goats, only because there are no data on the relationship between fecal nutrient and digestibility for cattle and horses, particularly in Mongolia. OMD was estimated based on fecal CP following the equation established for sheep on similar steppe condition of Inner Mongolia (Wang et al., 2009):


$$OMD\left(\%\right)=0.899-0.644*\exp\left(-0.5774*\frac{fecal\;CP\left(\frac{g}{kg}OM\right)}{100}\right)$$

### Analyses of soil chemistry

We sampled top soil only (between 2 and 5 cm depth), and mixed samples from 4–5 spots within our 10 m ×10 m vegetation plots. We did not use volumetric sampling but simply collected disturbed samples. Soil samples were also dried in the lab at 70 °C for 48 hours, then sieved through a 2 mm coarse screen, using fine material only for further analysis. We measured pH (soil: water ratio = 1:5) and electric conductivity (EC) in a soil water suspension, using a pH electrode after the suspension stayed still for 24 h. Soil samples were prepared for CN analysis in the same way as for biomass samples described above. We used the Olsen P method (Sims, 2000) to extract the amount of plant available phosphorus (aP). Phosphorus contents in the soil extracts were measured by spectrometry (ICP-OES, Institute of Soil Science, Hannover University). The content of inorganic C, i.e., carbonate, was first assessed with a quick test using 10% HCl, and we further analyzed samples which showed significant reactions using a calcimeter following Scheibler’s method (ON L 1084-99, 1999). Contents of organic C contents were then calculated by deducting inorganic C contents from total C content. A subsample of approximately 1 g of air-dried soil was oven-dried at 105 °C for 24 h to determine rest water in the soil. Contents of C, N and aP were corrected for rest water content, and expressed based on oven-dried soil.

### Assessment of climatic factors

We extracted “bioclimatic” variables (average for the years of 1970-2000, spatial resolution of ca. 1 km^2^) from Worldclim V2.1 (https://worldclim.org/data/worldclim21.html) (Fick and Hijmans, 2017) based on the coordinates of each study site. Climate data for each core site was calculated as the mean values from all sampled plots. Variables included mean annual temperature (MAT, BIO1) and mean annual precipitation sum (MAP, BIO12), precipitation of warmest quarter (BIO18), as well as precipitation seasonality (variation of monthly precipitation expressed as coefficient of variation; BIO15), temperature seasonality (standard deviation ×100; BIO4) and temperature annual range (BIO5-BIO6). Worldclim data is relatively old and may not represent the recent climate conditions due to evident climate change in Mongolia, but spatial data for the past 10 years are not available. However, we calculated MAP and MAT of 2010–2021 based on monthly precipitation and temperature measurements at the ten meteorological stations (http://www.tsag-agaar.gov.mn/) where our study core sites are close to; compared them with Worldclim data, and found both datasets being strongly correlated (MAP: *r* = 0.75, *p* < 0.001, MAT: *r* = 0.83, *p* < 0.001). Climate conditions between two sampling years did not differ much in terms of average precipitation and temperature (MAP: 254 mm in 2019 and 235 mm in 2020, MAT: 2.1 °C in 2019 and 1.8 °C in 2020).

### Quantification of disturbance factors

We included three proxies of livestock grazing impact. First, to estimate potential grazing pressure in the surveyed area for each site, we extracted numbers of livestock for the respective bagh, where our vegetation plots locate, from 2012 to 2021 from the National Statistical Office of Mongolia (NSO, 2025), and converted these into Mongolian sheep units (MSU) according to the NSO standard (1 horse − 7 MSU, 1 cattle – 6 MSU, 1 camel – 5 MSU, 1 goat – 0.9 MSU and 1 sheep – 1 MSU). Second, we also estimated the distance to settlement centers (dis2SC) from the surveyed vegetation plot to represent the gradient of local disturbance caused by human and livestock activity. We expected that as dis2SC increases, the level of livestock grazing decreases. We could not use well-designed grazing manipulation experiments with strict controls in the current study because they are not feasible at such large spatial scale. Last, to quantify the current livestock grazing intensity, we also estimated the total cover of dung (DungC) for main livestock groups on a 10 m × 10 m plot, locating next to the small plot where biomass samples were collected.

### Statistics

We considered climate (MAP, MAT), disturbance (dis2SC) and soil carbon-to-nitrogen ratio (soCN) as potential direct drivers of standing biomass and forage quality (CWM.N, CPY, OMD and t_water indicated by bold font in **Table 2**). Additionally, we conducted exploratory Principal Component Analyses (PCA) on all predictors and plant indicators to check the correlation structure of these variables and decide whether other environmental factors should be further considered, if they show different patterns from the interested ones. We then fitted those selected predictors on each of the single selected indicators of forage quality using Linear Mixed Models (LMM), always with “season” as fixed effects and “site” as a random factor. For each response, we started with a full model considering all selected predictors as fixed effects. We only considered the interaction between precipitation and disturbance, as we expected grazing effects to be more pronounced under relatively moist conditions (Wesche et al., 2010; Ahlborn et al., 2020). Other interactions were ignored because we did not have a respective hypotheses and we wanted to keep model complexity in range. We then progressively removed the least significant predictors until we only had the intercept in the model. Finally, the respective models were compared using ANOVA, and we used the least complex model, where further removal of predictors resulted in significant loss of exploratory power. We then recalculated this parsimonious model and took significances and slopes from that. All predictors fitted in the models were standardized, i.e., scaled to zero mean – unit variance. Model assumptions were checked by visual inspection of residual plots for the most parsimonious models. We also tested the LMM for core sites and satellite sites separately. Because we found no major difference in results, we opted for LMM with pooled data.

**Table 2:** Geographical location and climatic factors, soil chemistry, disturbance factors, characteristics of plant community and indicators of forage quality considered in the analysis.

Variables	Data range	Abbreviation
Geographical location and climatic factors		
Latitude (^0^)	45.26–49.32	Lat
Longitude (^0^)	105.90–118.48	Lon
Elevation (m, a.s.l.)	632–1713	Elev
**Mean annual precipitation total (BIO12, mm)**	178–339	**MAP**
**Mean annual temperature (BIO1, ^0^C)**	-2.2–2.3	**MAT**
Precipitation of warmest quarter (BIO18, mm)	131–246	Pwarm
Temperature seasonality (standard deviation ×100; BIO4)	1392.5–1632.3	SeasonTvar
Precipitation seasonality (coefficient of variation of monthly precipitation; BIO15, %)	95.1–128.9	SeasonP
Temperature annual range (BIO5–BIO6, ^0^C)	49.2–56.9	TyRange
Soil Chemistry		
Soil pH	5.9–7.7	pH
Soil electric conductivity	0.03–0.38	EC
Soil organic carbon (%)	0.7–5.1	soC
Soil total nitrogen (%)	0.1–0.5	soN
Plant available phosphorous (mg·kg^-^¹)	3.9–32.9	aP
**Soil carbon–to–nitrogen ratio (C/N)**	5.5–11.2	**soCN**
Disturbance factors		
**Distance to permanent settlement center (km)**	2.3–127.0	**dis2SC**
Livestock number at the *bagh* level in Mongolian sheep units (×1000)	9.1–175.4	MSU
Estimate of livestock dung cover (%)	0–7	DungC
Characteristics of plant community		
Species richness	3–28	sR
Total vegetation cover (%)	2–90	tCov
**Current standing biomass (kg·ha^-^¹)**	48.2–1791.3	**AGB**
Indicators of forage quality		
Nitrogen content of graminoids (%)	0.8–3.9	grN
Nitrogen content of forbs (%)	0.9–4.1	fN
**Community–weighted nitrogen content (%)**	0.8–3.8	**CWM.N**
Mean crude protein content (%)	5.2–23.8	CP
**Mean crude protein yield (kg·ha^-^¹)**	8.9–201.1	**CPY**
**Organic matter digestibility based on dung of sheep/goats (%)**	55.2–83.8	**OMD**
**Total water content in fresh biomass (%)**	7.3–81.0	**t_water**
Water content in graminoids (%)	3.9–98.1	gr_water
Water content in forbs (%)	3.2–93.3	f_water

Bold text indicates predictors and forage indicators selected based on PCA analyses for further fitting of linear mixed models − see text in method session for details.

All analyses were performed in R (v 4.5.2; R Core Team, 2025) with the packages geosphere (Hijmans, 2024) for distance estimation, vegan (Oksanen et al., 2020) for PCA, multcomp (Hothorn et al., 2008) for multiple comparison, lme4 (Bates et al., 2015), emmeans (Lenth and Piaskowski, 2026) and lmerTest (Kuznetsova et al., 2017) for LMM, ggplot2 (Wickham, 2016) and factoextra (Kassambara and Mundt, 2020) for visualization.

## Results

### Selection of forage indicators and predictors

**Figure 3:**
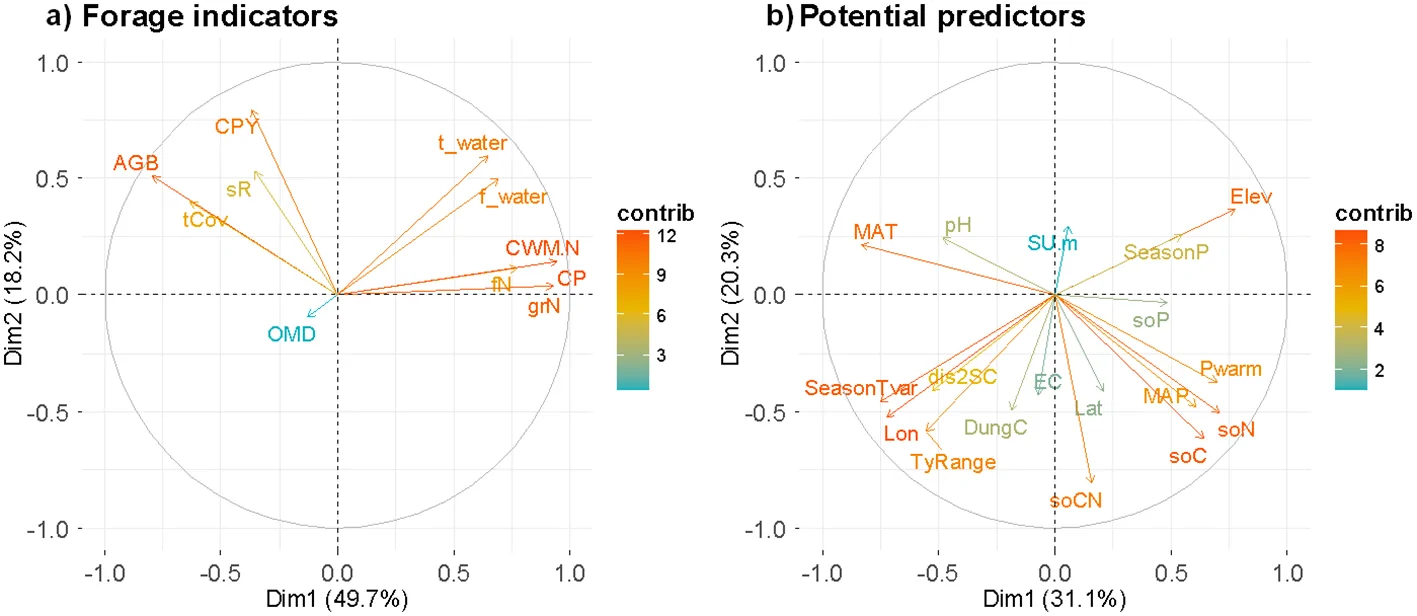
PCA analyses on **(A)** forage indicators and **(B)** potential predictors (for abbreviations see **Table 2**). **(A)** PC1 and PC2 together explained 68% and 51% of the total variance all forage indicators and predictors, respectively.

Exploratory PCA analyses on forage indicators (**Figure 3a**) resulted in a first axis closely associated with N contents of major functional groups, i.e., graminoids (grN), forbs (fN), and at the community-level (CWM.N), and a second axis closely related to crude protein yield (CPY), current standing biomass (AGB), and species richness (sR). Additionally, there were modest positive correlations between AGB and total vegetation cover (tCov, **Supplementary Figure 1**), as well as between total water content (t_water) and water content of forbs (f_water) and graminoids (gr_water). Digestibility (OMD) was not correlated with any of these indicators, and we thus kept CWM.N, CPY, OMD and t_water as response variables.

In PCA on environmental factors (**Figure 3b**) MAT and soil C-N ratio (soCN) explained most of the first two axes, respectively. Mean annual precipitation was correlated to Pwarm, soil organic carbon (soC) and total nitrogen (soN), while Dis2SC positively correlated to temperature seasonality, temperature annual range and longitude. We thus retained soCN, MAP, MAT and dis2SC as predictors for further analyses.

### Characteristics of plant richness and coverage, and of forage quality

**Table 3** presents means, standard deviations, and comparisons for all plant indicators across the three seasons. Within 1 m × 1 m quadrats, total vegetation cover remained similar across three seasons and species richness was only lower in spring than in summer. Current standing biomass in spring was only half of those from summer and autumn. Nitrogen contents of graminoids and community-weighted means declined from spring, to summer and autumn. N contents of forbs were similar in summer and spring, but lower in autumn. Yields of CP were lowest in spring, peaked in summer and declined to again in autumn. Digestibility was similar in summer and autumn, but lower in spring. Total water content and the water content of graminoids were highest in summer, followed by spring and autumn. However, the water content of forbs was similar in spring and summer, but only half of that in autumn.

**Table 3:** Summary (mean ± standard deviation, sd) of total vegetation cover, species richness, current standing biomass from the 1 m × 1 m quadrats, and indicators of forage quality for three seasons.

Indicators	Spring		Summer		Autumn	
Total vegetation cover [%]	35.0 ± 16.7	a	41.7 ± 13.2	a	41.9 ± 15.7	a
Species richness	9 ± 4	a	11 ± 4	b	10 ± 4	ab
Current standing biomass (AGB) [kg·ha^-^¹]	334.6 ± 187.0	a	866.9 ± 400.6	b	875.3 ± 285.0	b
N content of graminoids [%]	2.4 ± 0.5	a	2.0 ± 0.6	b	1.4 ± 0.4	c
N content of forbs [%]	2.6 ± 0.5	a	2.4 ± 0.6	a	1.8 ± 0.7	b
Community-weighted mean of N content [%]	2.4 ± 0.4	a	2.1 ± 0.6	b	1.5 ± 0.4	c
Mean crude protein content [%]	14.9 ± 2.8	a	13.0 ± 3.5	b	9.4 ± 2.5	c
Mean crude protein yield [kg·ha^-^¹]	48.4 ± 27.0	a	104.0 ± 37.7	b	77.8 ± 20.5	c
Organic matter digestibility [%]	59.6 ± 2.8	a	63.9 ± 5.2	b	63.7 ± 2.8	b
Total water content [%]	44.7 ± 11.9	a	54.1 ± 8.8	b	30.3 ± 15.1	c
Water content in forbs [%]	61.3 ± 13.8	a	62.8 ± 9.7	a	35.9 ± 20.8	b
Water content in graminoids [%]	38.7 ± 15.5	a	47.2 ± 12.3	b	26.5 ± 13.7	c

Different letters for the same plant indicator represent differences among seasons at a significance level of *p* < 0.05 based on LMM results.

### Drivers of forage quality

Fitting LMM showed that MAP and disturbance controlled forage quality (**Table 4**). Mean annual precipitation had negative impact on community–weighted N contents (CWM.N), but positive impact on yield of CP as AGB largely determined CP yield. Disturbance had only negative impact on CP yield. Mean annual temperature was positively related to total water content. However, no interactive impact of precipitation and disturbance was found on any indicator of forage quality.

**Table 4:** Summary of estimates and significances for abiotic (MAP, MAT) and biotic (dis2SC) predictors on current standing biomass (AGB) and indicators of forage quality in three seasons (all predictors scaled to zero mean – unit variance).

Indicators		Intercept	MAP	MAT	Dis2SC	Sp-Su	Sp-Au	Su-Au
AGB	Estimate	289.2***	162.6***		75.5*	-594.6***	570.6***	21.4
CWM.N	Estimate	2.4***	-0.19***			0.4***	0.9***	0.5***
CPY	Estimate	47.6***	9.5**		7.6*	57.9***	29.3***	28.2***
OMD	Estimate	0.60***				0.03**	0.04***	0.01
t_water	Estimate	44.8***		2.5*		8.0*	-15.4***	23.4***

Values were taken from the most parsimonious LMM models, which were achieved by model selection based on Likelihood Ratio Test, and are given only for those predictors that were still significant in most parsimonious models. Stars indicate significance levels (***< 0.001, **< 0.01, *< 0.05, ^•^< 0.1). Abbreviations for predictors and indicators of forage quality were explained in **Table 2**. Pairwise comparisons between seasons are noted as Sp-Su (spring minus summer), Sp-Au (spring minus autumn) and Su-Au (summer minus autumn).

**Figure 4:**
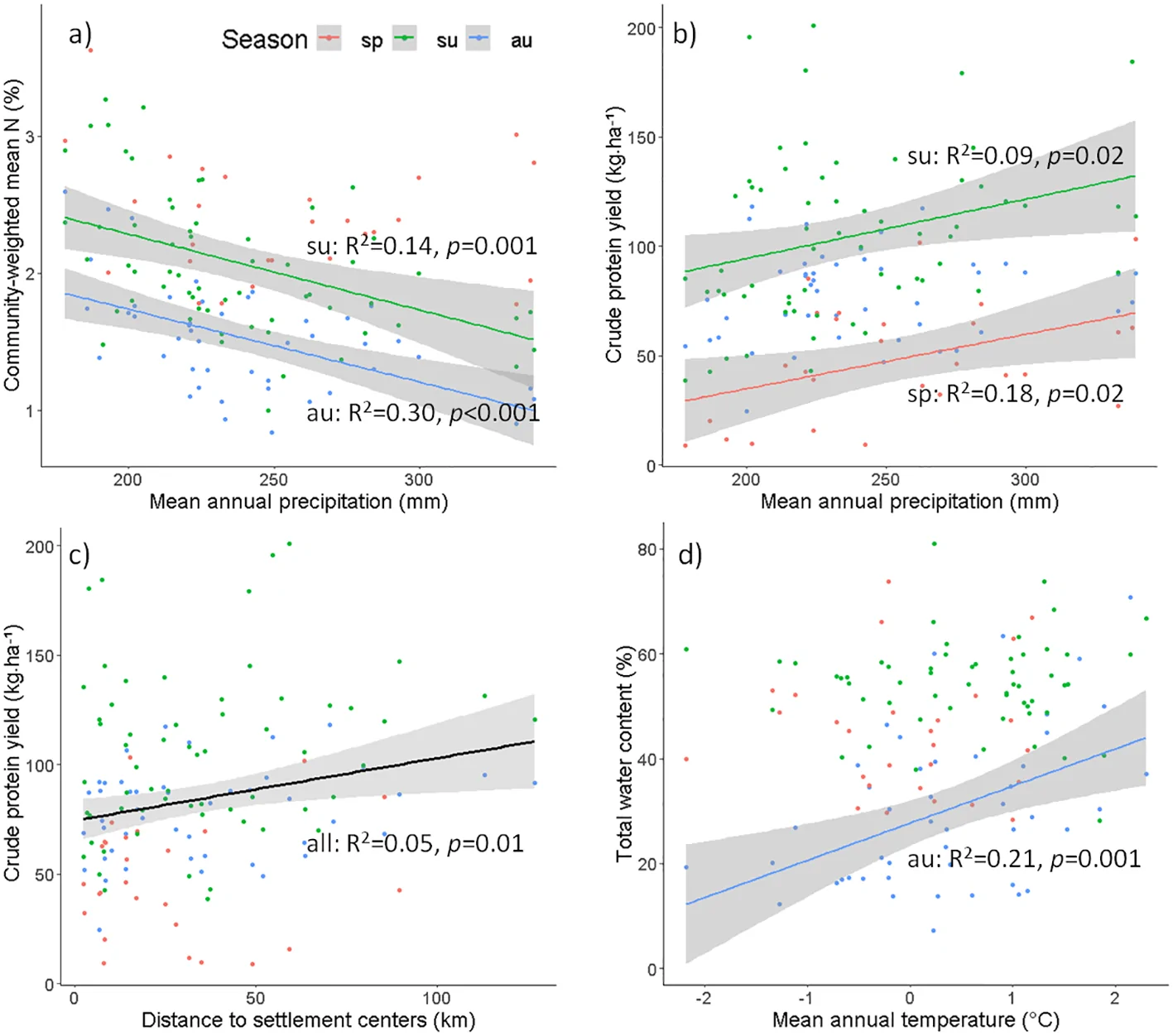
Patterns in key indicators of forage quality across three different seasons (sp = spring, su = summer, au = autumn). **(a)** community-weighted mean N contents, **(b)** crude protein yield along the gradient of mean annual precipitation, **(c)** crude protein yield along the distance to settlement centers, and **d)** total water content along the gradient of mean annual temperature. The shaded area shows the 95% confidence interval. Predictors are taken from those significant ones based on LMM results in **Table 4**, but separated correlation lines for corresponding seasons are only drawn here when season-wised Pearson correlations are significant. Details of correlation coefficients and significance levels are shown in **Supplementary Table 2**. For **(c)**, black correlation lines are only drawn for all seasons pooled together.

Moreover, effects of predictors on indicators of forage quality were not always significant in all seasons (**Supplementary Table 2**). A negative impact of MAP on CWM.N was shown only in summer and autumn (**Figure 4a**), while a positive impact of MAP on CP yield was shown only in spring and summer (**Figure 4b**). Disturbance reduced CP yields when three seasons are pooled together (**Figure 4c**). Total water content in autumn increased with MAT (**Figure 4d**).

## Discussion

### Seasonal dynamic of forage quality

We present the first large scale assessment of forage quality of eastern Mongolian steppe, and are among the first to cover extensive spatial and temporal gradients. With respect to seasonal dynamics, our results showed that plant N/CP contents at the community level, and the major functional groups, i.e., graminoids and forbs, declined significantly from spring to summer then to autumn. In the early growing season, plants have higher N but lower fiber contents, and once growth ceases, maturation and lignification processes begin towards the end of the growing season (Ren et al., 2016a), thus reducing N contents. In comparison with the few studies on forage quality in Mongolia, CP content from our spring samples in typical steppes (15%) was higher than those reported in mountain steppes (14%), but lower than those in mountain meadows (18%, Damiran and Sainkhuu, 2016). Our samples had a higher mean summer CP content (13%) than those from central Mongolia (12%, Oyundelger et al., 2025), but were lower than those of wild gazelle forage in eastern Mongolia (22%, Olson et al., 2010). These differences between vegetation types and regions are possibly caused by variations in plant community composition, as shown by large scale study in Chinese grasslands (Shi et al., 2013).

Mean crude protein yield (CPY) showed a different pattern from CP content, declining from summer to autumn, and with lowest values in spring, which was the same as the seasonal pattern of AGB. The lower CP yield in spring highlights the importance of considering spring values into assessments of carrying capacity, as a sole focus on higher summer CPY might lead to overstocking. The CPY values in summer (between 38 and 201 kg·ha^-^¹) from our study region are comparable to values reported from a typical steppe site in Inner Mongolia, i.e., 150–200 kg·ha^-^¹; the somewhat lower values may reflect differences in grazing intensity (Ren et al., 2016b). Although CPY in our study region was primarily controlled by AGB, overall forage quality may not increase when AGB increases because there was a decrease of digestibility as AGB increased, at least for autumn (**Supplementary Figure 2**). This is probably due to the increase of fiber content from dominating grasses (**Supplementary Table 1**).

Our estimates of digestibility (OMD) based on dung samples of sheep and goats were similar in summer and autumn (64%), but significantly lower in spring (60%). This is unexpected because young plant tissues are supposed to have higher CP contents and lower fiber contents, thus spring samples have higher CP and lower NDF (neutral detergent fiber) contents (Jiang et al., 2002; Schönbach et al., 2009). Our spring forage samples indeed had a higher CP content, but a lower digestibility. The only comparable studies on seasonal change of digestibility of ingested organic matter in Inner Mongolia did not include early growing season material (Schiborra, 2008; Müller et al., 2014). Others used an enzymatic method to measure *in vitro*-OMD of forage on offer, and showed reduced digestibility from early June to September at a typical steppe site in Inner Mongolia (Schönbach et al., 2009). Our results are based on forage ingested, which may differ from forage on offer, depending on whether the forage on offer is abundant or limited, and if animals select their diet or not (Schiborra, 2008). In spring, available forage amount is often limited, thus livestock consume small amounts of fresh forage along with larger amounts of senescent material (Murray et al., 1996), which has lower digestibility than living matter (Bumb et al., 2018). Additionally, when we collected spring samples, the weather was dry and AGB as well as water content of fresh biomass were both low, at least at certain study sites. Drought may reduce forage quality by increasing fiber and lignin contents (Schönbach et al., 2012; Habermann et al., 2019). Moreover, plant functional composition may differ among seasons. We found highest shares of biomass and coverage of graminoids in spring (**Supplementary Table 1**). Grasses have higher NDF content than forbs in spring and summer (Jiang et al., 2002), thus grass-dominated samples in spring potentially could have lower digestibility. Finally, water contents in fresh biomass were similar in spring and summer (ca. 62%), but much lower in autumn, showing that plants begin to dry out in autumn.

### Opposite precipitation impact: positive on CP yield, but negative on N content

Not surprisingly, we found precipitation positively controlling AGB, which has been shown in studies of *in situ* community biomass production (Ma et al., 2008; Nakano et al., 2020) and modeled primary productivity both in northern China (Liu et al., 2021; Wang et al., 2024b) and Mongolia (Bayarsaikhan et al., 2020; Rentsenduger et al., 2025). As the CPY was largely depending on AGB, precipitation showed similar positive impact on CPY, which is in line with the results from typical steppes in Inner Mongolia (Ren et al., 2016b). However, plant N content was negatively affected by precipitation, particularly in summer and autumn. This mirrors the general trend of higher leaf N contents in arid environments compared to wet tropical forests (Wright et al., 2004). High biomass N contents have been reported from dry Mongolian steppes before (Ronnenberg and Wesche, 2011), and negative correlations between CP content and MAP were also found on the eastern Qinghai-Tibetan Plateau (Wu et al., 2025). Moister conditions tend to accelerate plant growth resulting in dilution of N content, while plants in arid areas are efficient with respect N capturing (Ronnenberg and Wesche, 2011). They may reduce leaf expansion (limiting carbon assimilation) but invest in conserving N in their leaves to compensate for water scarcity and nutrient limitation (Han et al., 2025; Yan et al., 2025), Additionally, precipitation can affect N/CP contents indirectly by altering plant community composition. Studies have shown that grass/sedge dominated communities have lower CP contents than forb-dominated communities (Wu et al., 2025), because grasses and dicots differ in initial forage quality and rate of maturation (Carlsson, 2018). We found indeed that as MAP increased, the share of cover of graminoids within 10 m ×10 m vegetation survey plots in summer increased (*r* = 0.26, *p* = 0.04), and CP contents decreased (*r* = -0.22, *p* = 0.08).

### Negative impact of local disturbance on crude protein yield (CPY)

Local disturbance negatively affected AGB, particularly in spring and summer, which is in line with results from a typical steppe site in Inner Mongolia (Schönbach et al., 2009, 2012). However, we did not find disturbance impact on plant N/CP content. Previous studies showed positive impact of grazing on CP content in Inner Mongolia (Schönbach et al., 2009; Hoffmann et al., 2016; Ren et al., 2016a) and also in alpine grasslands of Tibet (Yao et al., 2022). Plant functional group composition may affect forage quality because forbs generally have higher forage quality than grasses (Bruinenberg et al., 2002; Duru et al., 2008), and the presence of legume species often promotes forage quality (Suter et al., 2015). We did not find a significant impact of disturbance on any functional group in terms of shares of coverage or biomass within 1 m × 1 m plot (**Supplementary Table 3**).

Crude protein yield (CPY) declined also with intensified grazing, which corresponds to the previous result that heavy grazing decreased annual forage productivity and nutrition yield such as CPY (Yao et al., 2022). The negative impact of grazing on CPY is largely because of the overriding impact of AGB on CPY. Moreover, a more detailed survey on plant functional composition in 10 m × 10 m plots in summer suggested modest increases in shares of coverage of shrubs (mostly legume species *Caragana* spp.) as well as forb legume species as distance to settlement center increased (**Supplementary Figure 3**). This may also contribute to the higher CPY at lower grazing levels.

### Climate impact on water content in fresh biomass

In arid environments or situations where access to drinking water is limited, high water content in forage could reduce the dependence of animals on walking to water holes (Squires, 1981). However, too much water in forage (>85%) may also limit dry-matter intake of animals (Lyons et al., 1999). Our samples showed 45%, 54%, and 30% mean water contents in spring, summer, and autumn, respectively. We only found a positive correlation between total water content and MAT, particularly for autumn biomass samples. This may reflect a phenological effect of delayed plant senescence because of warmer temperatures, in accordance with results from the manipulated experiment, which suggested that the temperature sensitivity of leaf senescence is 3–5 times larger in autumn than in summer (Wang et al., 2023).

However, we did not observe a correlation between water content in fresh biomass with precipitation, indicating that intrinsic factors such as plant morphology and physiology may be more important in determining how much water can be actually utilized by plants. The ability of adjusting osmotic potential to maintain turgor and physiological function under water stress differs among plant functional groups. Grasses use highly negative osmotic potentials to absorb water, while dicots have more inelastic tissues that rely on rapid turgor loss to adapt to dry conditions (Krasser and Kalapos, 2000). Abundant grasses (*Leymus chinensis* and *Cleistogenes squarrosa*) in Inner Mongolian typical steppes were shown to maintain 50-60% leaf water content under different soil water conditions (Chen et al., 2005). Our samples comprised a mixture of leaves, stems and even reproductive organs of plants, so leave-stem ratio and maturity stage of plants make it more complicated to explain the pattern of water content.

### No response in digestibility

Digestibility was invariant to any factor we tested. A previous study from meadow steppes in Inner Mongolia showed higher digestibility under heavy grazing (Hou et al., 2023), but these authors directly calculated digestibility by “1 – Fecal dry matter/Feed intake”. Another study from a typical steppe suggested no grazing impact on digestibility estimated by the same method as ours (Müller, 2009). Like other aspects of forage quality, digestibility is also related to forage characteristics, such as diet composition, maturity stage, plant morphology (leaf-to-stem ratio), and the fiber and lignin contents. Grazing could decrease the share of lignified plant parts and promote the re-growth of new tissue, leading to increased CP content and decreased lignification, thus enhancing digestibility (Schönbach et al., 2009). However, intensified grazing could also increase acid detergent lignin and thereby reduce digestibility (Glindemann et al., 2009). Our relatively wide range of digestibility values indicates a diverse diet composition, which is supported by pronounced heterogeneity in plant species compositions in the study region (Jäschke et al., 2026). Therefore, variability in diet and the chemical composition of the diet may lead to complex responses of digestibility to grazing.

### Impact of soil chemistry

Although we did not find any direct impact of soil C-N ratio on forage indicators, our data suggested an indirect impact of soil on plant functional group composition. Soil C-N ratio negatively affected share of forbs in terms of coverage and biomass (**Supplementary Table 3**). As a rough proxy of soil nitrogen availability, soil C-N ratios in our study region ranged from 5.5 to 11.2, which is within the range of 6.4-18.8 from a recent report of soil C-N ratio from dry steppe grassland in eastern Mongolia (Khulan et al., 2025), and 4.5-17.1 from Inner Mongolian grasslands (Chen et al., 2003). Our data suggested a marginal increase of autumn AGB and CPY as soil C-N ratio increased (**Supplementary Figure 4**; **Supplementary Table 2**), which is in accordance with the results from a recent large-scale study on grasslands in northern China, suggesting productivity increases with soil C-N ratio when soil C-N value <15 (Zhang et al., 2025). The possible reason for a non-significant impact in summer could be a relative high activity of microbes under high temperature and moisture conditions, which compete with plants for N (Schrama et al., 2013). Cooling temperatures may reduce microbial activity, resulting in higher N availability to plants (Zhang et al., 2017).

## Conclusion

The opposite impact of MAP on plant N contents and CP yield suggests a trade-off between forage quantity and quality, which is often explained by the nutrient dilution effect, i.e., the decrease in the concentration of nutritional elements in plant tissue – arising from an increase in the mass of carbohydrates. It is thus crucial to consider this effect and integrate forage quality, such as CP yield into estimates of grassland carrying capacity. Since forage availability varies among seasons, the respective lowest values are critical for sustainable use of rangelands, and should be taken with great concern rather than only evaluating data from peak biomass in summer. Additionally, forage quality needs to be assessed by different facets because these may response differently to grazing and climate factors. Lower CP yield under intensified grazing close to permanent settlement centers gives reason for concern. Pasture rotation and reduction of livestock numbers around settlement centers are recommended to allow recovery of forage quality in eastern Mongolia steppes. As soil C-N ratio affected plant functional group composition, and thus indirectly have impact on forage quality, soil conditions need to be also monitored to prevent potential soil degradation.

## Data Availability

The datasets presented in this study can be found in online repositories. The names of the repository/repositories and accession number(s) can be found below: https://doi.pangaea.de/10.1594/PANGAEA.993583.
