# Supplementary material for: Divergent responses of forage quality indicators to environmental drivers in Eastern Mongolia
Source: Front Plant Sci. 2026 Aug 24;17:1860377. doi: 10.3389/fpls.2026.1860377 (PMC13564203; doi:10.3389/fpls.2026.1860377)
Supplement: Supplementary file 1 [file SupplementaryFile1.docx]

Supplementary Figure 1. Pearson correlation between total vegetation cover and the current standing biomass in 1 m ×1 m quadrats for different seasons (spring: *r* = 0.33, *p* = 0.02; summer: *r* = 0.38, *p* < 0.001; autumn: *r* = 0.38, *p* < 0.001).
