# Supplementary material for: Divergent responses of forage quality indicators to environmental drivers in Eastern Mongolia
Source: Front Plant Sci. 2026 Aug 24;17:1860377. doi: 10.3389/fpls.2026.1860377 (PMC13564203; doi:10.3389/fpls.2026.1860377)
Supplement: Supplementary file 2 [file SupplementaryFile2.docx]

Supplementary Figure 2. Pearson correlation between current standing biomass and organic matter digestibility for autumn (*r* = -0.35, *p* = 0.02). Correlations in spring and summer were non-significant.
