# Supplementary material for: Divergent responses of forage quality indicators to environmental drivers in Eastern Mongolia
Source: Front Plant Sci. 2026 Aug 24;17:1860377. doi: 10.3389/fpls.2026.1860377 (PMC13564203; doi:10.3389/fpls.2026.1860377)
Supplement: Supplementary file 3 [file SupplementaryFile3.docx]

Supplementary Figure 3. Pearson correlations between distance to settlement center and a) share of coverage of shrubs (*r* = 0.26, *p* = 0.04), b) share of coverage of legumes (*r* = 0.21, *p* = 0.1) based on vegetation survey data at 10 m × 10 m plots.
