# Supplementary material for: Divergent responses of forage quality indicators to environmental drivers in Eastern Mongolia
Source: Front Plant Sci. 2026 Aug 24;17:1860377. doi: 10.3389/fpls.2026.1860377 (PMC13564203; doi:10.3389/fpls.2026.1860377)
Supplement: Supplementary file 4 [file SupplementaryFile4.docx]

Supplementary Figure 4.Pearson correlation between soil C-N ratio and crude protein yield for spring (ns) summer (ns) and autumn (*r* = 0.28, *p* = 0.02)
