# Supplementary material for: Divergent responses of forage quality indicators to environmental drivers in Eastern Mongolia
Source: Front Plant Sci. 2026 Aug 24;17:1860377. doi: 10.3389/fpls.2026.1860377 (PMC13564203; doi:10.3389/fpls.2026.1860377)
Supplement: Supplementary file 5 [file Table1.docx]

Supplementary Table 1. Shares (mean ± sd) of biomass, cover of different functional groups in total dry biomass, and total cover of the small plot where we collected biomass samples split by different seasons.

|  | Share of biomass (%) | | | | Share of coverage (%) | | | |
| --- | --- | --- | --- | --- | --- | --- | --- | --- |
|  | forb | graminoid | shrub | subshrub | forb | Graminoid | shrub | subshrub |
| Spring | 18.0±17.3 | 58.2±25.8 | 11.7±23.0 | 11.8±16.3 | 21.3±18.9 | 78.4±12.5 | 8.2±19.5 | 12±14.3 |
| Summer | 32.4±22.2 | 52.5±22.8 | 3.6±8.7 | 10.6±16.3 | 38.8±19.8 | 48.9±20.9 | 2.4±4.4 | 8.6±15.6 |
| Autumn | 27.6±19.2 | 60.3±19.1 | 2.7±5.3 | 9.5±14.4 | 28.3±19.8 | 74.5±13.6 | 2.8±4.7 | 10.6±15.6 |
|  |  |  |  |  |  |  |  |  |
