# Supplementary material for: Divergent responses of forage quality indicators to environmental drivers in Eastern Mongolia
Source: Front Plant Sci. 2026 Aug 24;17:1860377. doi: 10.3389/fpls.2026.1860377 (PMC13564203; doi:10.3389/fpls.2026.1860377)
Supplement: Supplementary file 6 [file Table2.docx]

Supplementary Table 2. Pearson correlation coefficients and significance levels (*** < 0.001, ** < 0.01, * < 0.05, ^•^ < 0.1) between current standing biomass, selected indicators of forage quality and relevant predictors as indicated by LMM results (from Table 4) for spring, summer, and autumn, respectively, as well as for all data across seasons. Abbreviations for predictors and indicators were explained in details in Table 2.

| Predictor | Indicator | Seasons | | | | | | Across seasons | |
| --- | --- | --- | --- | --- | --- | --- | --- | --- | --- |
|  |  | Spring | | Summer | | Autumn | |  |  |
| MAP | AGB | 0.44 | * | 0.44 | *** | 0.52 | *** | 0.29 | *** |
|  | CWM.N | -0.17 | ns | -0.37 | ** | -0.55 | *** | -0.65 | *** |
|  | CPY | 0.43 | * | 0.27 | * | 0.12 | ns | 0.80 | *** |
| soCN | AGB | 0.25 | ns | 0.18 | ns | 0.27 | ^•^ | 0.16 | ^•^ |
|  | CWM.N | -0.03 | ns | -0.14 | ns | -0.21 |  | -0.14 | ns |
|  | CPY | 0.26 | ns | 0.17 | ns | 0.28 | * | 0.12 | ns |
|  | t_water | 0.08 | * | -0.13 | ns | -0.22 | * | -0.16 | ^•^ |
| dis2SC | AGB | 0.04 |  | 0.16 |  | 0.12 | ns | 0.19 | * |
|  | CWM.N | -0.24 | ns | -0.07 | ns | -0.04 | * | -0.08 | ns |
|  | CPY | 0.07 |  | 0.20 |  | 0.24 | ns | 0.22 | ** |
| MAT | t_water | 0.004 | ns | -0.06 | ns | 0.46 | *** | 0.19 | * |
