# Supplementary material for: Divergent responses of forage quality indicators to environmental drivers in Eastern Mongolia
Source: Front Plant Sci. 2026 Aug 24;17:1860377. doi: 10.3389/fpls.2026.1860377 (PMC13564203; doi:10.3389/fpls.2026.1860377)
Supplement: Supplementary file 7 [file Table3.docx]

Supplementary Table 3. Summary of estimates and significances for effects of abiotic (MAP, MAT, soCN) and biotic (dis2SC) predictors on functional composition at the 1m × 1m plot level, in terms of share of coverage and biomass in three seasons (all predictors scaled to zero mean – unit variance). Values were taken from the most parsimonious LMM models, which were achieved by model selection based on Likelihood Ratio Test, and are given only for those predictors that were still significant in most parsimonious models. Stars indicate significance levels (*** < 0.001, ** < 0.01, * < 0.05, ^•^ < 0.1). Abbreviations for predictors and indicators of forage availability were explained in Table1. Season.Sp (spring) and Season.Su (summer) represent the comparison against autumn.

| **Indicator** |  | **Intercept** | **MAP** | **MAT** | **soCN** | **Dis2SC** | **MAP:dis2SC** | **Season.Su** | **Season.Au** |
| --- | --- | --- | --- | --- | --- | --- | --- | --- | --- |
| **Share of coverage** | | | | | | | | | |
| Forbs | Estimate | 21.89** |  |  | -4.94** |  |  | 16.31*** |  |
| Graminoids | Estimate | 77.84*** |  |  |  | -2.73^•^ |  | -28.83*** |  |
| Subshrubs | Estimate | 9.99*** | 3.81* |  |  |  |  |  |  |
| Shrubs | Estimate | 8.31*** | -4.05*** | *** |  |  | -2.51^•^ | -9.66** | -6.17** |
| **Share of biomass** | | | | | | | | | |
| Forbs | Estimate | 20.97*** |  |  | -6.52** |  |  | 10.84** | 8.04* |
| Graminoids | Estimate |  |  |  |  |  |  |  |  |
| Subshrubs | Estimate | 11.86*** | -5.34*** |  |  |  | -3.35^•^ | -9.54*** | -10.03*** |
| Shrubs | Estimate | 10.25*** |  | ** | 2.78^•^ |  |  |  |  |
